# Protective immunity in mice vaccinated with a novel elastase-1 significantly decreases *Trichinella spiralis* fecundity and infection

**DOI:** 10.1186/s13567-020-00767-z

**Published:** 2020-03-14

**Authors:** Xin Zhuo Zhang, Xiang Yuan Sun, Ying Bai, Yan Yan Song, Chen Xi Hu, Xiangrui Li, Jing Cui, Zhong Quan Wang

**Affiliations:** 1 College of Veterinary Medicine, Nanjing Agricultural University, Nanjing, 210095 China; 2 Department of Parasitology, Medical College, Zhengzhou University, Zhengzhou, 450052 China

## Abstract

*Trichinella spiralis* is an important foodborne parasitic nematode that represents an enormous threat to the food safety of pork meat. The development of a preventive vaccine is valuable for the prevention and control of *Trichinella* infection in domestic pigs to ensure pork safety. Elastase is a trypsin-like serine protease that hydrolyzes the host’s diverse tissue components and participates in parasite penetration, and it might be a novel vaccine target molecule. The aim of this study was to assess the protective immunity produced by vaccination with a novel *Trichinella spiralis* elastase-1 (TsE) in a mouse model. The results demonstrate that subcutaneous vaccination of mice with rTsE elicited a systemic humoral response (high levels of serum IgG and subclass IgG1/IgG2a and IgA) and significant local enteral mucosal sIgA responses. Anti-rTsE IgG recognized the native TsE at the cuticle, stichosome of intestinal infective larvae and adult worm (AW), and intrauterine embryos of female AW. The rTsE vaccination also produced a systemic and local mixed Th1/Th2 response, as demonstrated by clear elevation levels of Th1 cytokines (IFN-γ, IL-2) and Th2 cytokines (IL-4, IL-10) after spleen, mesenteric lymph node and Peyer’s patch cells from immunized mice were stimulated with rTsE. The immunized mice exhibited a 52.19% reduction in enteral AW and a 64.06% reduction in muscle larvae after challenge infection. The immune response triggered by rTsE vaccination protected enteral mucosa from larval intrusion, suppressed larval development and reduced female fecundity. The results indicate that TsE may represent a novel target molecule for anti-*T. spiralis* vaccines.

## Introduction

*Trichinella spiralis* is an important foodborne parasitic nematode that is distributed worldwide in over 150 kinds of mammals [1]. Human *T. spiralis* infection principally results from ingestion of the encapsulated infective larvae present in raw or uncooked meat and meat products. Domestic pigs are the crucial infection source of human *T. spiralis* infection in China and other developing countries [2–5]. From 2004 to 2009, 14 trichinellosis outbreaks owing to infected domestic pork and wild boar meat were reported in the Chinese mainland [6]. As a considerable quantity of pork is consumed around the world, *T. spiralis* infection in domestic pigs represents a severe risk to public health and a notable hazard to pork meat safety [5, 7]. Therefore, it is necessary to develop a preventive vaccine to block *Trichinella* infection in domestic swine and transmission from swine to humans [8–10].

After the infected meat is ingested, *T. spiralis* muscle larvae (ML) are released from their capsules with the aid of gastric fluid digestion and develop into intestinal infective larvae (IIL) after being exposed to enteral contents or bile [11, 12]. The IIL intrudes the enteral epithelium and grows to adult worm (AW) stages after molting four times. The adult males and females mate, and pregnant females yield the next generation of larvae (newborn larvae, NBL). The NBL enters the blood circulation, invades the host’s striated muscles and forms encapsulated larvae to complete its lifecycle [13]. The intestinal epithelium is the primary native protective screen against *T. spiralis* intrusion and the principal interaction place of the host and the parasite [14, 15], but the mechanism of intrusion of the intestinal epithelium by *T. spiralis* larvae has not been fully elucidated [16, 17]. The excretion/secretion (ES) products of *T. spiralis* IIL larvae, which are first exposed to intestinal epithelium cells (IECs), are likely to have a crucial effect on larval intrusion and elicit the enteral mucosal response [18, 19]. In our previous studies, some serine proteases have been identified in *T. spiralis* IIL and AW ES products by immunoproteomics [20, 21]. When *T. spiralis* IIL larvae were cocultured with an IEC monolayer, the IIL intruded the monolayer and generated secretory serine proteases and entered the IEC [22, 23]. Furthermore, the expression levels of serine proteases at the IIL stage were evidently higher than those at the ML stage [24]. As a result, *T. spiralis* serine proteases might facilitate larval intrusion into the enteral epithelium and aid the nematode in establishing intestinal infection [25–27]. Therefore, serine proteases are promising candidate vaccine targets against *T. spiralis* enteral phase worms.

In this study, a novel elastase gene of *T. spiralis* (TsE, GenBank accession no. EFV56917.1) was retrieved from the draft genome of *T. spiralis* [28]. The elastase, which belongs to the serine protease family, was cloned, expressed and purified in our laboratory. Bioinformatic analysis results revealed that the complete TsE cDNA sequence was 1350 bp encoding 449 amino acids with a 47.3 kDa. The TsE carried a functional domain at 38-314 aa. TsE was highly expressed at the *T. spiralis* ML and IIL worm phases [29]. Recombinant TsE (rTsE) promoted larval intrusion into the IEC monolayer, whereas anti-rTsE serum and RNAi suppressed larval invasion. The objective of this study was to assess the immune protection produced by rTsE immunization in a model of BALB/c mice.

Although complete Freund’s adjuvant (CFA) is one of the most commonly used adjuvants for experimental antibody generation, its clinical application has been limited due to the side effects and local lesions that it causes. The series of Montanide incomplete Seppic adjuvants (ISAs) are new-generation adjuvants of water-in-oil emulsions (w/o) after being mixed with antigens, which have favorable adjuvant characteristics for eliciting a long-term and strong immune response [30]. The ISA contains an injectable pharmaceutical mineral oil and a refined emulsifier originating from mannitol, purified vegetable oleic acid, and copolymers of methacrylic acid. Furthermore, subcutaneous inoculation has the advantages of easy injection, rapid absorption, and high immune efficacy. Therefore, BALB/c mice were immunized by subcutaneous inoculation with the rTsE formulated with ISA201 adjuvant of Montanide ISA series in the present study.

## Materials and methods

### Parasites and experimental animals

The *T. spiralis* strain (ISS534) utilized in the present study was collected from a domestic pig in mainland China and passaged in our department [31]. Four-week-old female BALB/c mice were purchased from the Experimental Animal Center of Henan Province (Zhengzhou, China) and raised in specific pathogen-free conditions of individual ventilated cages (IVCs, Suzhou, China) [32].

### Preparation of rTsE

The complete TsE gene was cloned, and the recombinant expression plasmid pQE-80L/TsE was transformed into *Escherichia coli* BL21 (Novagen, USA). Expression of rTsE protein was induced using 1 mM IPTG at 37 °C for 6 h [33]. rTsE was purified using Ni–NTA-Sefinose resin (Sangon Biotech, China) in our laboratory and identified by anti-rTsE serum and *T. spiralis*-infected mouse sera by Western blot analysis.

### Immunization of mice

A total of 210 mice were randomly divided into three groups (70 animals per group). Each mouse was subcutaneously immunized by using 20 µg rTsE emulsified with Montanide ISA 201 adjuvant (Seppic, France) and boosted three times with rTsE with ISA 201 at a 4-week interval for the first immunization and at a two-week interval for the second and third boosts. The control group was administered ISA 201 adjuvant or PBS alone according to the same protocol as the immunization group [9, 30]. Approximately 100 μL of blood was taken from each mouse tail prior to immunization and at 4, 6, 8 and 10 weeks after immunization. Individual serum sample was kept at – 80 °C until use [34].

### Detection of antibody responses to rTsE

Serum levels of anti-rTsE antibodies (total IgG, IgG1, IgG2a, and IgA) in all vaccinated mice were tested by ELISA with rTsE [35, 36]. Briefly, the ELISA plate was coated using 1 μg/mL rTsE for total IgG and 2 μg/mL rTsE for IgG1, IgG2a and IgA at 37 °C for 2 h. Skimmed milk at a 5% concentration was used for blocking at 37 °C for 1 h. Different dilutions of mouse sera (1:100 for detecting IgG, IgG1 and IgG2a; 1:50 for detecting IgA) were added and incubated at 37 °C for 2 h. Following washes with TBS-0.5% Tween 20 (TBST), 1:10 000 dilutions of HRP-conjugated anti-mouse IgG, IgG1 or IgG2a, and 1:5000 dilutions of HRP-conjugated anti-mouse IgA were added and incubated for 1 h at 37 °C [37]. Plates were developed with o-phenylenediamine dihydrochloride (OPD; Sigma-Aldrich) plus H_2_O_2_, and the reaction was terminated by using 2 M H_2_SO_4_. The absorbance at 492 nm was assayed using a microplate reader (Tecan, Schweiz, Switzerland) [38, 39].

### Assessment of total IgA and secretory anti-TsE IgA in intestinal washes

To measure total and TsE-specific secretory IgA (sIgA) in enteral fluid, intestinal washing was prepared [10, 40]. Briefly, a 20-cm-long small intestine segment was cut, and the enteral interior was eluted three times using 1 mL of PBS with 1% protease inhibitor (Sangon Biotech, Shanghai, China). The flushing fluid was collected and centrifuged at 10,000*g* for 5 min, and the supernatants were harvested [31]. Total intestinal sIgA was determined by a conventional sandwich ELISA as previously described [41]. TsE-specific sIgA was measured by a routine indirect ELISA with 1 μg/mL of rTsE as described [42, 43]. The coloration with OPD and assay of the absorbance at 492 nm were carried out as described before [44].

### Recognition of native TsE in worms at different *T. spiralis* stages by immunofluorescence test (IFT)

The identification of native TsE in *T. spiralis* diverse lifecycle stage worms was performed using an immunofluorescence test (IFT) with worm cross-sections [45, 46]. The ML were obtained by artificial digestion of skeletal muscles from experimentally *T. spiralis*-infected mice at 42 days post-infection (dpi) [47]. IILs were recovered from the infected mouse intestine at 6 hpi [24], and adult worms (AWs) were collected from the intestine at 3 dpi [48]. The worms were embedded in paraffin, 3-µm-thick cross-sections were prepared, and IFT was carried out as previously reported [32, 49]. The sections were sealed at 37 °C for 1 h with 5% normal goat serum and probed by a 1:10 dilution of three different sera (anti-rTsE serum, infection serum and normal serum) at 37 °C for 1 h. Following three washes with PBS, the worm cross-section was stained at 37 °C for 1 h with FITC-anti-mouse IgG conjugate (1:100; Santa Cruz, USA) and observed under a fluorescence microscope (Olympus, Japan) [50, 51].

### Cytokine determination

To ascertain the TsE-specific cellular immunological response, the spleens, mesenteric lymph nodes (MLNs) and Peyer’s patches (PPs) were recovered from vaccinated mice at weeks 0, 4, 6, 8 and 10 following immunization. Spleen, MLN and PP were ground with complete RPMI-1640 medium (Gibco, Auckland, New Zealand), the pellets were obtained after centrifugation at 300*g* for 5 min, and the cells were separated as reported before [45, 52]. The cellular density was set to 2 × 10^6^ cells/mL in RPMI-1640 medium supplemented with 5% fetal bovine serum (FBS; Gibco), penicillin (100 U/mL) and streptomycin (100 μg/mL). The cells were stimulated using 10 μg/mL rTsE for 72 h, the supernatant was harvested, and four cytokines (IL-2, IL-4, IL-10 and IFN-γ) were determined by sandwich ELISA [9, 53]. The concentrations of four cytokines were presented as picograms per milliliter (pg/mL).

### Oral larva challenge infection and assessment of immune protection

To appraise the immune protection induced by immunization with rTsE, each vaccinated mouse was orally inoculated using 300 *T. spiralis* infective larvae 2 weeks after the fourth vaccination. The AWs were recovered from the small intestines of ten mice of each group at 7 dpi [54, 55]. The ML were collected from an additional ten mice at 42 dpi by digesting the infected mouse striated muscles [56]. Moreover, the length of adult females was measured by microscopy. Adult female reproduction capacity was also determined after being cultivated in RPMI-1640 culture medium for 72 h, the NBL yielded by each adult female was accounted for, and their length was examined [57]. The effectiveness of immune protection produced by TsE immunization was ascertained as the parasite reduction of enteral adult worms and larvae per gram (lpg) of striated muscles of immunized mice in comparison with those of the PBS group [58–60].

### Antibody-dependent cell-mediated cytotoxicity (ADCC) assay

In this study, anti-rTsE antibody cytotoxicity on the NBL was also conducted as previously reported [39, 61]. Forty NBLs were cocultivated for 48 h at 37 °C with 2 × 10^5^ mouse peritoneal exudate cells (PECs) in 96-well plates in RPMI-1640 medium containing anti-rTsE serum, infection serum and preimmune serum. Each assay was performed in triplicate. The larval viability after ADCC treatment was ascertained according to larval morphological characteristics and activity by microscopy. The live NBL were mobile and exhibited wriggling, whereas the dead worms were straight and immobile. The cytotoxicity was assessed as the percent of the dead worms to the total worms observed in each assay [50].

### Statistical analysis

Statistical analysis of all the data was conducted by using SPSS for Windows, version 20.0. The data are represented as the mean ± standard deviation. Differences among diverse groups were analyzed by one-way ANOVA. A Chi square test was applied to analyze the relationship between ADCC cytotoxicity and serum dilution/culture time. *P* < 0.05 was considered to be significant.

## Results

### Humoral immunological response to rTsE immunization

**Figure 1:**
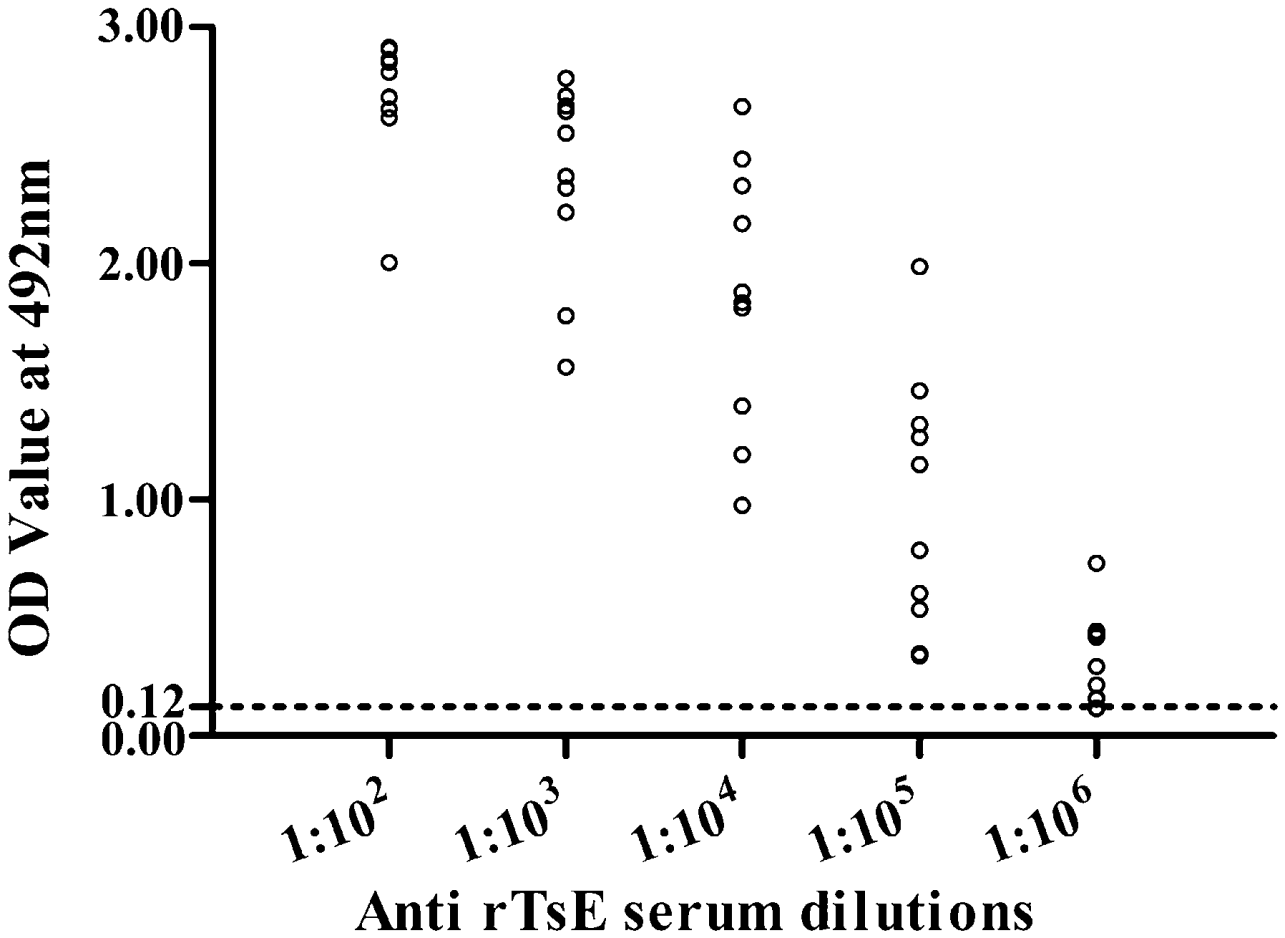
**Serum titers of anti-rTsE IgG antibody measured by rTsE-ELISA.** Thirty serum samples (1:100 dilution) from normal mice were measured as negative serum controls. The cut-off values (0.124) are shown with a dotted line.

**Figure 2:**
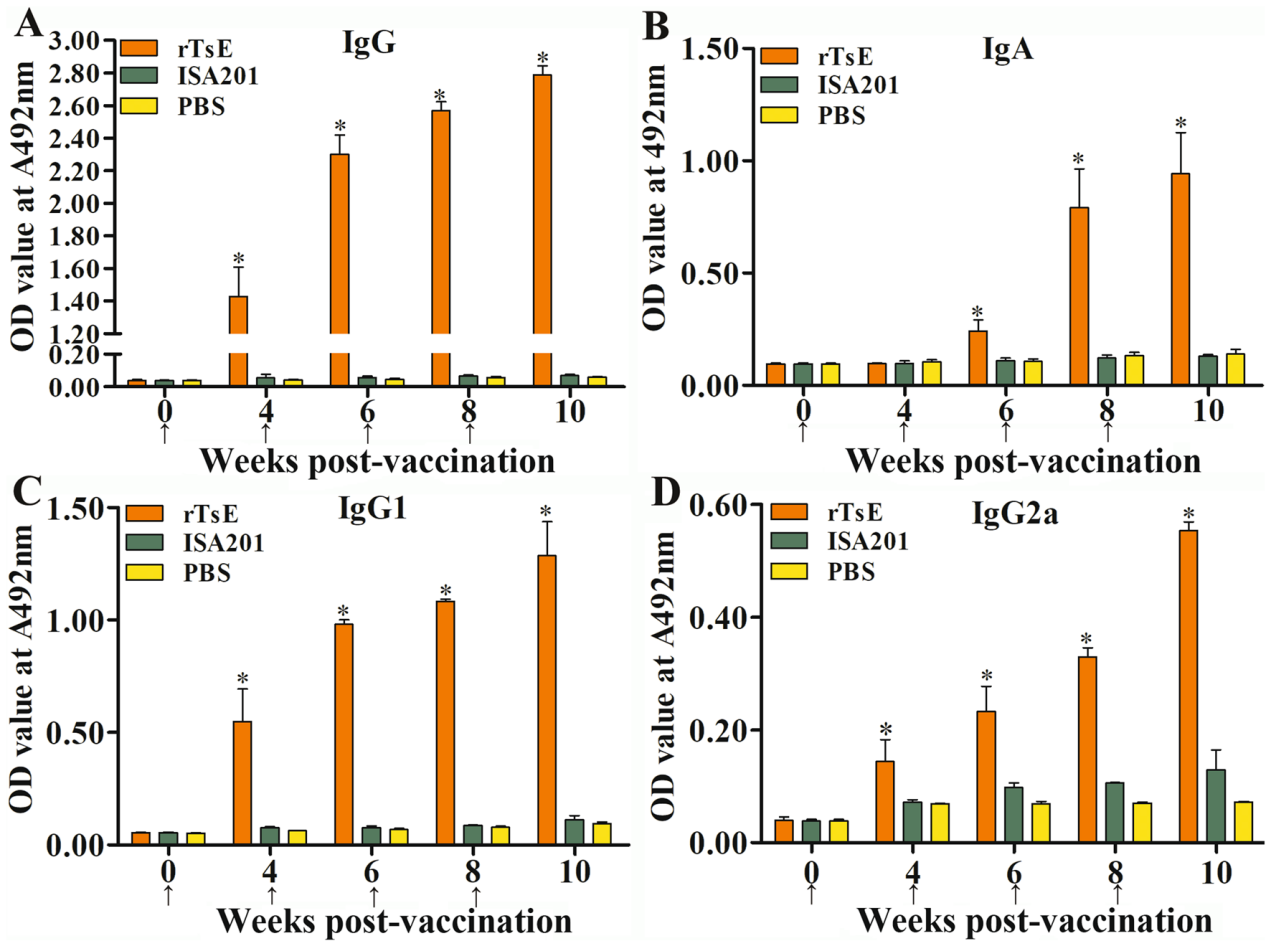
**Specific anti-rTsE antibody responses in mice vaccinated with rTsE.** Specific total IgG response was determined in rTsE-vaccinated mice or control mice (ISA 201 adjuvant and PBS) at various time intervals post-vaccination (**A**). **B** Specific IgA levels in vaccinated mice. rTsE-specific IgG1 (**C**) and IgG2a (**D**) subclass responses were also detected at various times post-vaccination. The OD value from each group is presented as the mean ± SD of antibody levels (*n* = 10). The vaccination time is indicated as an arrow (↑). *P < 0.05 compared with the ISA 201 adjuvant or PBS group.

The mice were vaccinated using rTsE four times, and the serum anti-rTsE antibody IgG titer at two weeks after the fourth vaccination was measured by ELISA. The anti-rTsE IgG level in mice vaccinated with rTsE was prominently elevated, and the mean antibody titer reached 1:10 000 after the fourth vaccination (Figure 1), indicating that rTsE was a strong immunogenic protein. However, no anti-rTsE IgG antibody responses were detected in mice inoculated with ISA 201 alone or PBS (Figure 2A). The IgG1 level at 4, 6, 8 and 10 weeks post-vaccination was obviously higher than the IgG2a level (Figures 2C, D) (week 4: t = 2.564, *P *= 0.027; week 6: t = 17.553, *P* < 0.0001; week 8: t = 33.294, *P* < 0.0001; week 10: t = 26.911, *P* < 0.0001), suggesting that the dominant IgG subclass elicited by rTsE immunization was IgG1. Nonetheless, the IgA response was also triggered after immunization (Figure 2B).

### Intestinal mucosal immune response elicited by TsE immunization

**Figure 3:**
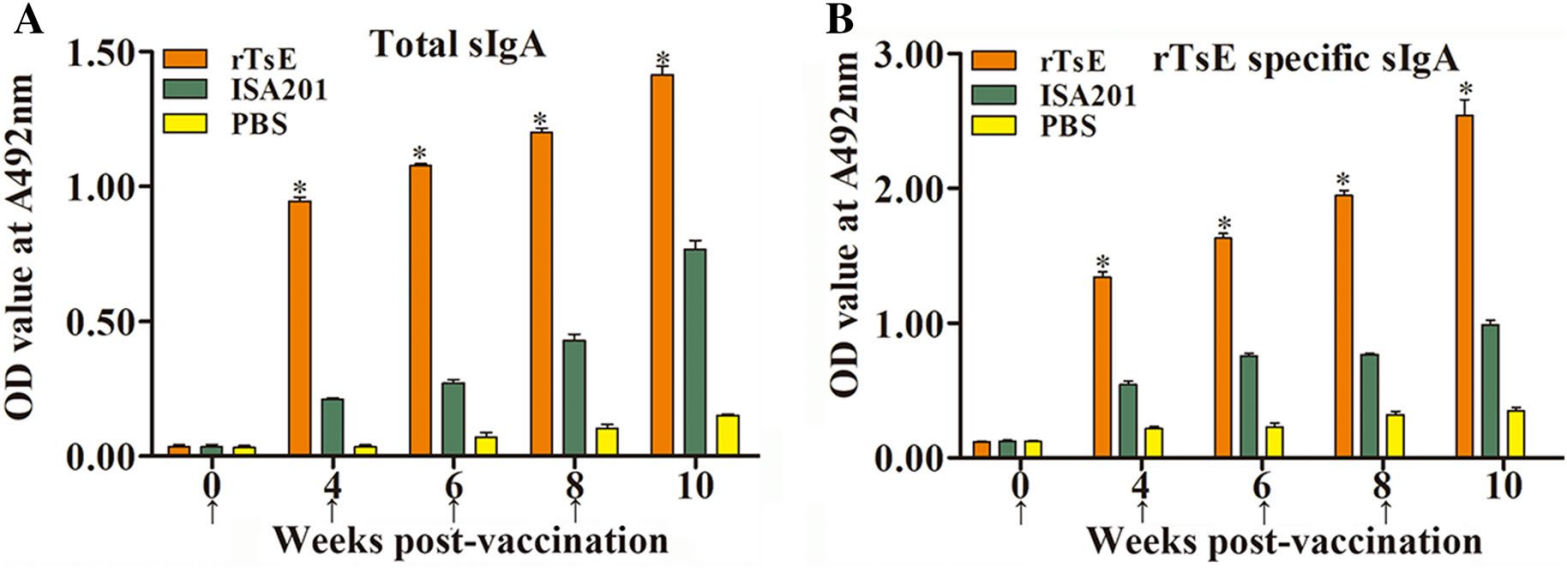
**Levels of total intestinal sIgA (A) and TsE-specific sIgA (B) in intestinal washing of vaccinated mice.** The data are shown as the mean OD values ± SD for 10 mice per group. TsE-specific sIgA was undetectable in mice injected with only ISA 201 or PBS. The vaccination time is indicated as an arrow (↑). *P < 0.0001 compared with PBS and ISA 201 adjuvant group.

To assess the enteral mucosal sIgA response to rTsE immunization, total sIgA and TsE-specific sIgA were determined using ELISA. The results revealed that the total sIgA level was conspicuously elevated in rTsE-immunized mice compared to mice inoculated with only ISA 201 adjuvant (week 4: t = 44.572, *P* < 0.0001; week 6: t = 61.563, *P* < 0.0001; week 8: t = 28.126, *P* < 0.0001; week 10: t = 13.864, *P* < 0.0001) (Figure 3A). The levels of intestinal-specific anti-TsE sIgA in mice immunized with rTsE were also distinctly higher than those of mice injected with ISA 201 alone (week 4: t = 17.837, *P* < 0.0001; week 6: t = 22.582, *P* < 0.0001; week 8: t = 33.342, *P* < 0.0001; week 10: t = 12.691, *P* < 0.0001) (Figure 3B). No rTsE-specific mucosal sIgA was detected in mice injected with only PBS and ISA 201.

### Recognition of native TsE at diverse stage worms by IFT

**Figure 4:**
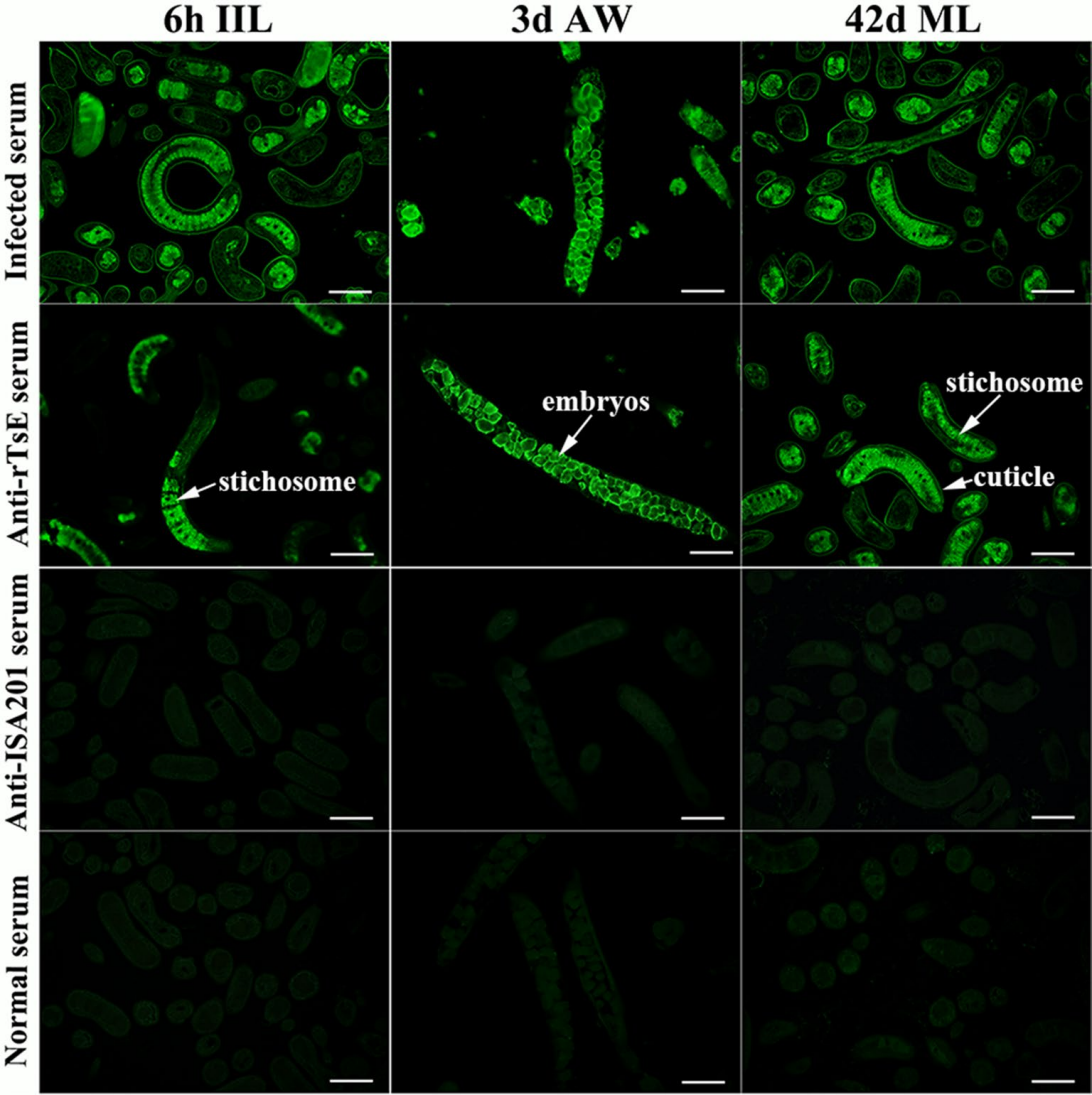
**Recognition of native TsE at diverse*T. spiralis*stage worms by IFT with serum from mice vaccinated by rTsE, alone ISA 201 adjuvant or PBS. Green fluorescence was located at the cuticle, stichosome of IIL and AW, and intrauterine embryos of female AW of this parasite.** The serum of mice inoculated using ISA 201 adjuvant alone and PBS did not identify any worm tissue components of the parasites. Scale bar = 100 μm.

The IFT results showed that anti-rTsE serum recognized the natural TsE on worm cross-sections, green fluorescence was primarily at the cuticle, at the stichosome of IIL and AW, and at intrauterine embryos of female adult worms (Figure 4). Serum from mice inoculated with only ISA 201 adjuvant and PBS did not identify any worm tissue component of the parasitic nematode.

### Cytokine response to rTsE vaccination

**Figure 5:**
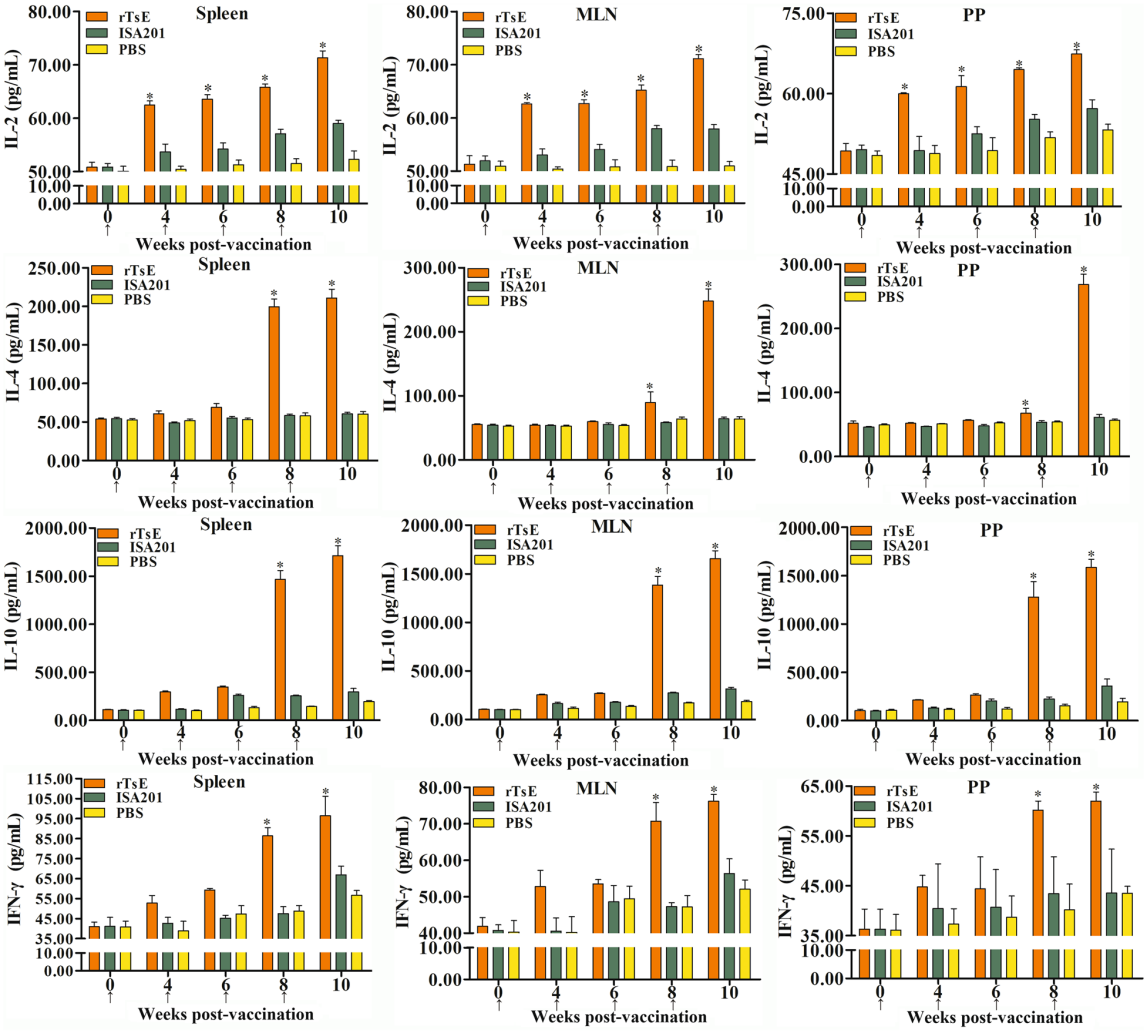
**Cytokines secreted by the spleen, mesenteric lymph nodes (MLNs) and Peyer’s patches (PPs) of mice vaccinated with rTsE at various times post-vaccination. **The concentrations of four cytokines (IFN-γ, IL-2, IL-4, and IL-10) were determined in the supernatant after spleen, MLN and PP cells were stimulated by 10 μg rTsE for 72 h. The values are shown as the mean ± SD of ten animals/group. The vaccination time is indicated as an arrow (↑). *P < 0.0001 relative to the ISA201 and PBS control groups.

To assess the cytokine response elicited by rTsE vaccination, spleen, MLN and PP cells obtained from vaccinated mice were cultivated under rTsE stimulation. Supernatants were collected, and cytokine concentrations were measured using sandwich ELISA. The levels of Th1 (IFN-γ) and Th2 cytokines (IL-4, IL-10) clearly increased after the third immunization with rTsE compared with the ISA201 and PBS groups (*P* < 0.0001) (Figure 5). Moreover, the IL-2 levels were elevated earlier than those of the other three cytokines. Our results demonstrated that rTsE vaccination triggered mixed Th1/Th2 responses on the basis of specific IgG subclass responses and cytokine generation, suggesting that subcutaneous vaccination with rTsE triggered both systemic (spleen) and enteral mucosal local (MLN and PP) cellular immunological responses.

### Immune protection produced by rTsE immunization against *T. spirali* larval infection

**Figure 6:**
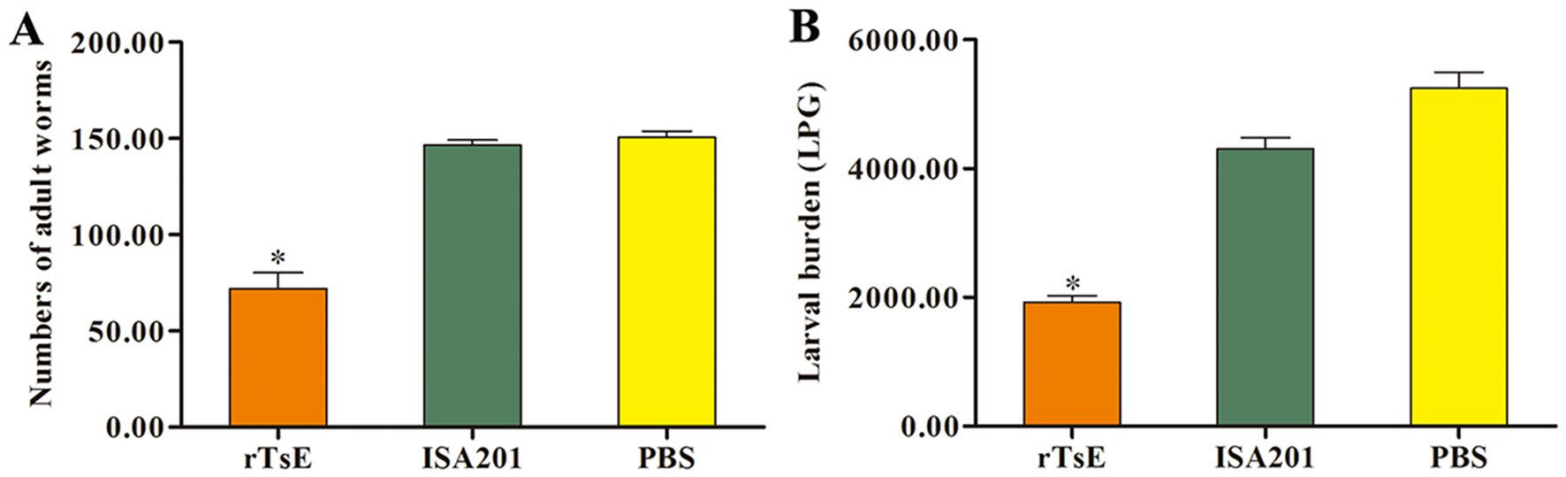
**Immune protection efficacy of rTsE immunization following oral infection with* T. spiralis* larvae in a mouse model. A** Intestinal adult worm burden; **B** Muscle larva burden (larvae per gram, lpg). The parasite burden is presented as the mean ± SD from rTsE-vaccinated mice, only ISA201 adjuvant and PBS group (*n* = 10). * *P* < 0.0001 compared to the ISA201 adjuvant and PBS groups.

Compared with PBS control mice, the rTsE-immunized mice showed a 52.19% decrease in enteral adults at 7 dpi (Figure 6A) and a 64.06% decrease in muscle larvae at 42 dpi (Figure 6B) after oral infection with 300 *T. spiralis* muscle larvae (*F*_adults_ = 11.330, *P* < 0.0001; *F*_larvae_ = 11.107, *P* < 0.0001).

**Figure 7:**
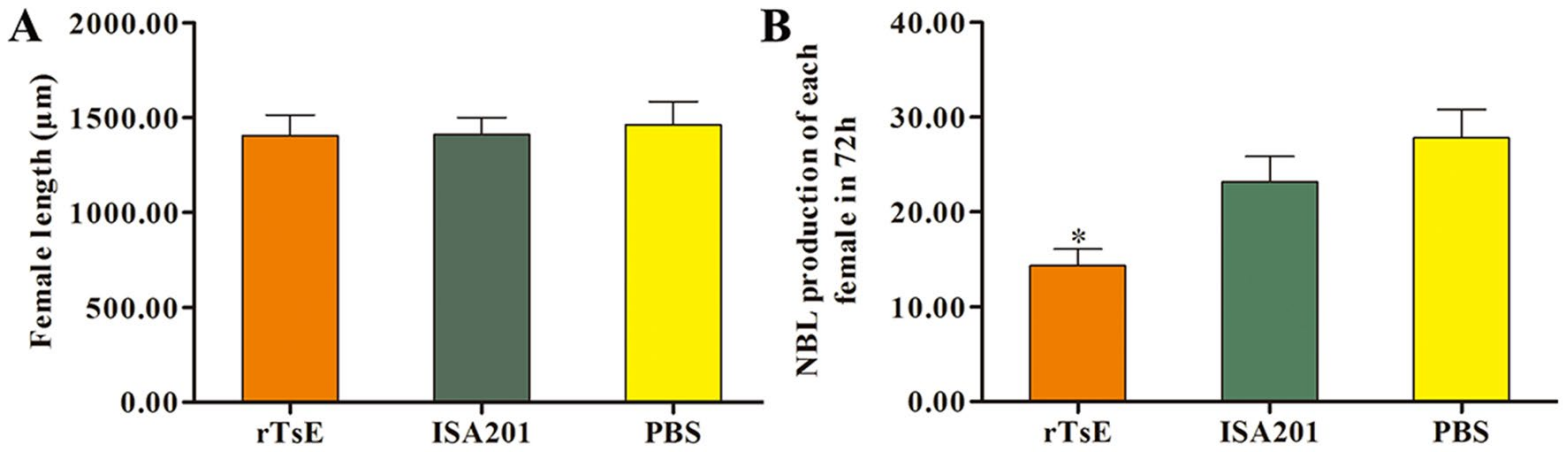
**Length and fecundity of T. spiralis female adults from rTsE-vaccinated mice at 7 days following larval challenge (n = 10). a** Female adult length; **b** Newborn larval production of each female in vitro for 72 h.

Additionally, the length of female adults of rTsE-immunized mice at 7 dpi had no statistically obvious difference compared with those from the ISA201 and PBS groups (*F* = 0.89, *P *> 0.05). However, the production of NBL yielded in vitro for 72 h by each female adult from rTsE-immunized mice was evidently inferior to that of the ISA201 and PBS groups (Figure 7) (*F* = 7.110, *P* < 0.01). The results indicated that immunization of mice with rTsE decreased the enteral adult burden and reduced adult female fecundity, thereby relieving the larval burden in skeletal muscles of immunized mice.

### Destruction of NBL by ADCC

**Figure 8:**
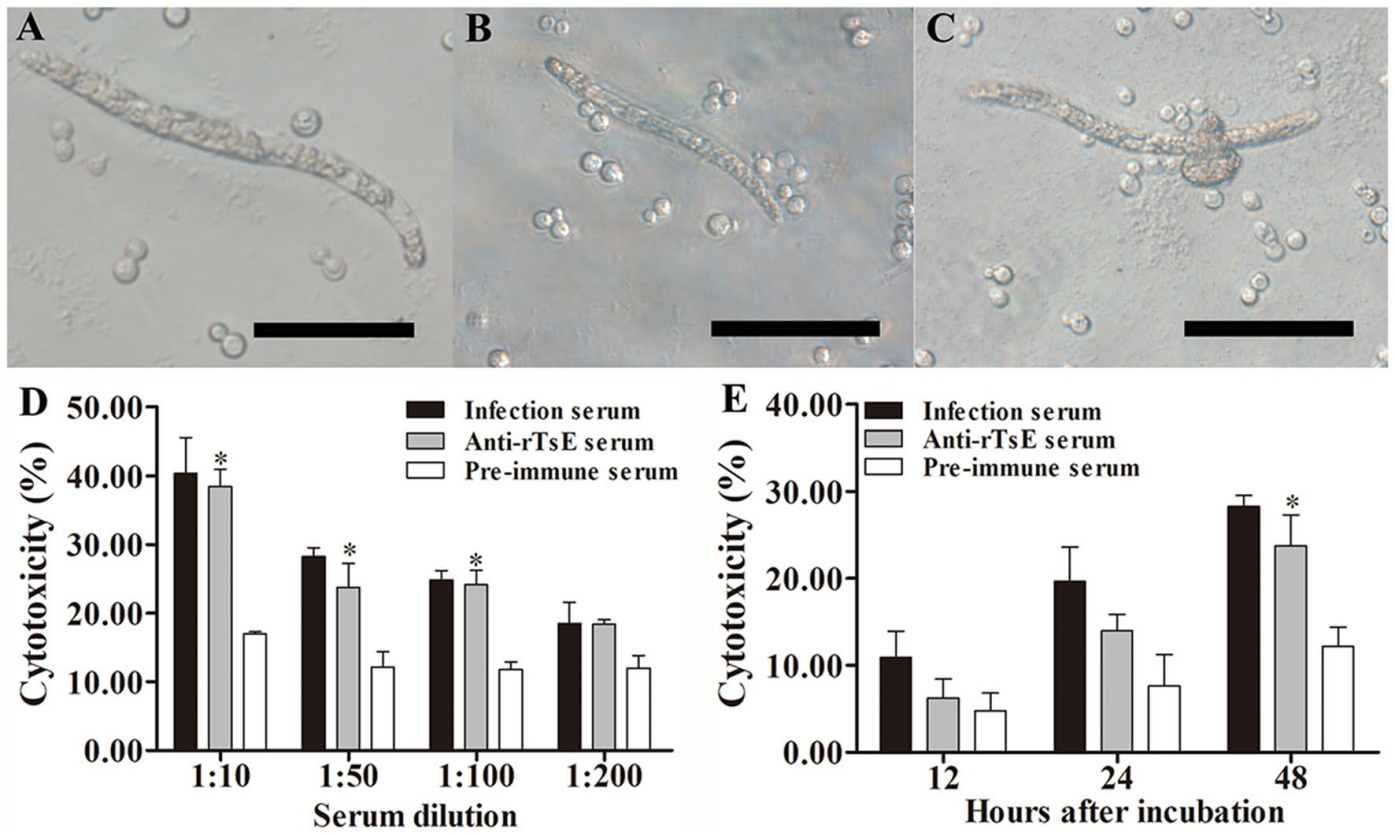
**Killing effects of ADCC on* T. spiralis* NBL.** The NBL were cocultivated with anti-rTsE serum and 2 × 10^5^ mouse peritoneal exudate cells (PECs) at 37 °C for 48 h. **A** Live NBL; **B** PEC adherence to NBL; **C** dead NBL. **D** Cytotoxicity was dose-dependent for anti-rTsE antibodies. **E** Cytotoxicity also increased with prolonged cultivation time. *P < 0.05 compared with the preimmune serum group. Scale bars: 50 μm.

The results of the ADCC test revealed that after being cultivated at 37 °C for 48 h, anti-rTsE serum mediated the attachment and destruction of the PECs to the NBL (Figure 8). While 1:10, 1:50 and 1:100 dilutions of anti-rTsE sera were supplemented, the ADCC led to a significant death of the NBL (38.45%, 24.15% and 23.76% cytotoxicity) compared to the NBL cocultivated in preimmune serum (17.00%, 12.18% and 11.77%) (t = 8.280, *P*_1:10_ < 0.01; t = 3.102 *P*_1:50_ < 0.05; t = 5.200, *P*_1:100_ < 0.017). The cytotoxicity was anti-rTsE antibody dose dependent (r = 0.898, *P* < 0.0001), and the cytotoxicity had a decreasing trend following the increase in serum dilutions (*F* = 44.582, *P* < 0.0001). There was also a clear correlation between cytotoxicity and culture time (r = 0.917, *P* < 0.01), and cytotoxicity showed an increasing trend with prolonged culture time (*F* = 33.420, *P* < 0.01).

## Discussion

Elastase is a trypsin-like serine protease that can hydrolyze the host’s diverse tissue components (e.g., fibronectin, laminin, type IV collagen, elastin). The elastases participate in parasite penetration, molting and digestion and may play a major role in worm intrusion of hosts [62]. In our previous study, TsE was cloned, expressed and identified, and it was likely a novel target molecule for preventive vaccines against *T. spiralis* infection. In this study, immunization of mice with the rTsE protein generated a specific serum antibody response against rTsE, and the anti-rTsE antibody IgG titer was 1:10 000, suggesting that rTsE had strong immunogenicity. Our results showed that anti-rTsE IgG from vaccinated mice recognized the native TsE at the cuticle, stichosome of this nematode, and intrauterine embryos of female AW. Subcutaneous vaccination with rTsE produced a systemic humoral immune response and significant local enteral mucosal sIgA responses. sIgA acts an important function in mucosal defense and might protect the intestinal epithelium from parasite intrusion [42, 63]. Anti-*T. spiralis* sIgA mediated intestinal adult expulsion from the gut, and passive transfer of the anti-*Trichinella* antibody IgA produced evident protection against larval challenge in mice [64].

Our results also indicated that subcutaneous immunization with rTsE induced the mixed Th1/Th2 response, as demonstrated by an obvious elevation of Th1 cytokines (IFN-γ, IL-2) and Th2 cytokines (IL-4, IL-10) after spleen, MLN and PP cells from immunized mice were stimulated with rTsE. The mixed Th1/Th2 response is crucial for immune protective efficacy against *Trichinella* infection [45]. In addition, the production of mucosal sIgA is Th2-dependent; in particular, IL-10 is a main cytokine that enhances the IgA response. In this study, the correlation of elevated enteral sIgA levels and high IL-10 levels demonstrate that the cytokine IL-10 enhances the enteral mucosal sIgA response.

Following *T. spiralis* challenge infection, the rTsE-immunized mice exhibited a 52.19% decrease in enteral adults at 7 dpi and a 64.06% decrease in muscle larvae at 42 dpi. The worm burden decrease observed in the present work is similar to those of previous studies [26, 32, 54]. The immune protective effect may be due to the production of a high level of specific anti-TsE IgG antibody, which neutralized the hydrolyzing ability of elastase. Anti-*Trichinella* IgG also binds to the cuticle of enteral stage worms and forms a cap-like immune complex in the parasite anterior end, which physically interdicts the worms’ direct contact with enteral epithelia, blocking worm intrusion of enteral mucosa and impairing worm growth and development [65]. Anti-*Trichinella* antibody IgG also participated in damage and destruction of newborn larvae through an ADCC pattern [66]. To ascertain the cytotoxicity of anti-TsE antibodies, an ADCC assay was also conducted in this study. The results indicated that TsE-specific antibody facilitated macrophage adherence and damage to the NBL, and ADCC cytotoxicity was dose-dependent for anti-TsE antibodies.

Although the length of enteral female adults from immunized mice was not significantly different from those from the ISA 201 or PBS group, the female fecundity (e.g., the NBL production/female in vitro for 72 h) was significantly inferior to that from ISA 201 or PBS control mice. Previous studies indicated that enteral sIgA suppressed the reproductive capacity of *T. spiralis* adult females [67]. Our results suggested that protective immunity induced by immunization with rTsE also depressed intestinal worm development and impaired the reproductive capacity of adult females [10, 43].

In recent years, some protein molecules from various *T. spiralis* lifecycle stages have been characterized and expressed, and their immune protection was also evaluated, such as paramyosin (Ts-Pmy) from an adult cDNA library [9], TspGST and fructose-1,6-bisphosphate aldolase (Ts-FBPA) from the *T. spiralis* draft genome with high expression at the ML stage [33, 68], Ts31 from the ML ES proteins [54], serine protease (TsSP) from IIL and ML surface proteins [55], and cathepsin B (TsCB) from the *T. spiralis* draft genome [56]. However, when these recombinant proteins were used for vaccinating mice, they exhibited only 36.2–53.50% ML reduction following *T. spiralis* challenge. In the present study, we ascertain the protective immunity induced by vaccination with a novel TsE protein. Since TsE is a secretory protein that is highly expressed at the *T. spiralis* intestinal invasive stage (IIL), TsE might be exposed early to the host’s intestinal mucosa and elicit the local immune response. Our results indicated that vaccination with rTsE induced significantly high levels of TsE-specific sIgA, which might facilitate adult worm expulsion from the intestine [64]. The immune protection (64.06% ML reduction) with this novel TsE vaccination was superior to those of the abovementioned other *T. spiralis* proteins as candidate vaccine target molecules. This study also established a foundation to develop polyvalent anti-*T. spiralis* vaccines in the future.

Trichinellosis principally results from the oral ingestion of infected animal meat. *Trichinella* is a multicellular enteral and tissue-lodging parasite that has a complicated lifecycle, and diverse *Trichinella* developmental phase worms have stage-specific antigens. Vaccination with a single *Trichinella* protein molecule or peptide merely elicited approximately 60% protection from larva challenge infection. In future studies, polyvalent vaccines should be developed and contain multiple protective antigenic epitopes from diverse *T. spiralis* developmental phases (IIL, AW, NBL and ML) to elicit both systemic/enteral local humoral and cellular immune responses. To acquire full immune protection, ideal polyvalent vaccines must prevent *Trichinella* infection and block the development of clinical trichinellosis at various lifecycle phases: interrupting IIL larval invasion of enteral epithelia, blocking IIL larval development to the adult stage, dislodging adult worms from the guts, interdicting or impeding NBL generation from enteral residual adults, killing escaping NBL to prevent their migration in blood and larval encapsulation in skeletal muscles [36, 69]. Hence, oral polyvalent vaccines against different *T. spiralis* life cycle phases need to be developed to interrupt *Trichinella* infection transmission among domestic food animals [9].

In conclusion, our results showed that subcutaneous vaccination of mice with a novel rTsE produced a systemic Th1/Th2 mixed response and local enteral mucosal sIgA response. The vaccinated mice showed significant immune protection, as demonstrated by a 52.19% reduction in enteral adults and a 64.06% reduction in muscle larvae following *T. spiralis* larval challenge. The immune responses triggered by rTsE vaccination also inhibited enteral worm development and reduced the reproductive capacity of adult females. The results indicated that TsE might be considered a novel target molecule for anti-*T. spiralis* vaccines.

## Abbreviations

ADCC: Antibody-dependent cell-mediated cytotoxicity
AW: Adult worms
BSA: Bovine serum albumin
ES: Excretion-secretion
IEC: Intestinal epithelial cells
IFT: Immunofluorescence test
IIL: Intestine infective larvae
lpg: Larvae per gram
ML: Muscle larvae
MLN: Mesenteric lymph nodes
NBL: Newborn larvae
OPD: o-Phenylenediamine dihydrochloride
PECs: Peritoneal exudate cells
PP: Peyer’s patches
rTsE: Recombinant TsE
sIgA: Secretory IgA
TBST: TBS-0.5% Tween 20
TsE: *T. spiralis* elastase-1
